# Increases in fecal testosterone precede increases in fecal dehydroepiandrosterone in wild male mountain gorillas (*Gorilla beringei beringei)*

**DOI:** 10.1007/s00360-026-01702-y

**Published:** 2026-07-27

**Authors:** Ella R. Brown, Rose Umuhoza, Carine Uwamahoro, Alliance Umwizerwa, Winnie Eckardt, Tara S. Stoinski, Michael D. Cherney, Richard J. Auchus, Stacy Rosenbaum

**Affiliations:** 1 Department of Anthropology, University of Michigan, Ann Arbor, USA; 2 Dian Fossey Gorilla Fund, Musanze, Rwanda; 3 Dian Fossey Gorilla Fund, Atlanta, USA; 4 Departments of Pharmacology and Internal Medicine, University of Michigan, Ann Arbor, USA

**Keywords:** Testosterone, DHEAS, Puberty, Adrenarche, Male development, Mountain gorillas

## Abstract

Testosterone (T) and dehydroepiandrosterone sulfate (DHEAS) are two steroids integral to male primate development. For humans, both are high in early infancy (for T, this is known as minipuberty) then rapidly decline. DHEAS then increases beginning in early childhood, resulting in a process known as adrenarche, which signals the maturation of the zona reticularis of the adrenal gland. T increases a few years thereafter, marking the beginning of puberty. These processes are relatively well-studied in humans, but data is lacking for the other great apes. Particularly for adrenarche, strikingly little is known about its distribution across the primate order. Here, we draw on a large sample of wild male mountain gorilla fecal samples collected over the full course of the gorilla lifespan to examine how the hormones T and DHEAS change with age. We use enzyme-linked immunosorbent assays to quantify metabolites of both hormones, and validate these assays using high-performance liquid chromatography–tandem mass spectrometry analyses. We find that both hormones are high in male gorilla infancy, and then decline before T rises again at 9.3 years of age. Surprisingly, DHEAS rises *afterwards* at 10.7 years old, showing a departure from the human developmental pattern. T then peaks at 18 years of age, while DHEAS plateaus at ~ 23 years of age. More research is needed to assess intra-order variation in primate hormonal development.

**Supplementary Information:**

The online version contains supplementary material available at https://doi.org/10.1007/s00360-026-01702-y.

## Introduction

The androgens testosterone (T) and dehydroepiandrosterone sulfate (DHEAS) are associated with hallmark milestones in human development (Auchus and Rainey 2004; Wood et al. 2019). While these developmental processes are relatively well-studied in humans, we know remarkably little about them in our closest relatives, the other extant great apes. Identifying human-like (or non-human-like) patterns in these hormones across development in other primate species will both aid our understanding of the evolutionary history of *Homo sapiens*’ unusually extended developmental timelines, and help us to better understand the physiology of these animals in their own right.

DHEAS, which is the sulfated form of the hormone DHEA, is an androgen produced by the zona reticularis (ZR; the innermost layer) of the adrenal cortex, and is the most abundant circulating steroid in the human body for both males and females (Xing et al. 2015). High circulating concentrations of DHEAS appear to be unique to primates (Conley et al. 2004; Norris and Carr 2013b), as it is either absent or occurs in only small amounts in non-primate mammals (Cutler et al. 1978; Schiebinger et al. 1981; van Weerden et al. 1992). The functions of DHEAS are numerous, although not completely understood: at minimum, DHEAS is a precursor to sex hormone production outside of the gonads (Labrie et al. 2001; Xing et al. 2015), acts as a neuroactive steroid (Maninger et al. 2009), and has been implicated in immune function (Leenstra et al. 2003; Prall et al. 2015). More speculatively, it may also be important in brain and socio-behavioral development and developmental plasticity (Brown et al. 2024; Campbell 2020; Cumberland et al. 2021; Del Giudice 2018).

During normal development, both male and female humans experience high DHEAS just after birth. This is a result of the fetal zone of the adrenal gland, which produces DHEAS in utero to be converted into estrogens that normally rise during pregnancy (Auchus and Rainey 2004). After birth, the fetal zone regresses via apoptosis, resulting in a drop in DHEAS in infancy and a period of quiescence in early childhood (Auchus and Rainey 2004; Del Giudice 2018). This drop in DHEAS is clearly not limited to humans: an initial decline in DHEAS during infancy has also been observed in macaques (Kemnitz et al. 2000; Conley et al. 2011), sooty mangabeys (Mann et al. 1983), baboons (Castracane et al. 1981; Ducsay et al. 1991), and chimpanzees (Sabbi et al. 2020). In humans, this is the time during which the other two layers of the adrenal cortex (the zona glomerulosa and the zona fasciculata) form (Kim and Choi 2020).

For humans, the ZR becomes morphologically detectable at ~ 4 years of age (Hui et al. 2009), while DHEA and its metabolites (which includes DHEAS) begin to increase exponentially at ~ 3 years of age (Palmert et al. 2001; Remer et al. 2005; Kushnir et al. 2010). Roughly two years before the onset of puberty (~ 6–8 years old for humans), the accelerated increase in DHEAS associated with the maturation of the ZR produces clinical manifestations in a process known as *adrenarche* (Auchus and Rainey 2004). Here, we will refer to this pattern of postnatal, pre-pubertal ZR formation and DHEAS production as “human-like” adrenarche. To date, apart from humans, human-like adrenarche has been most extensively documented in chimpanzees, based on adrenal and hormonal data collected across infancy into adulthood (Bernstein et al. 2012; Parker et al. 2014; Sabbi et al. 2020), and has also been noted in bonobos (Behringer et al. 2012). These observations have been cited as evidence that human-like adrenarche is perhaps a derived trait in only *Homo* and *Pan* (Bernstein et al. 2012; Campbell 2020). Most recently, however, postnatal, pre-pubertal increases in DHEAS have also been identified in four species of hylobatids (Hart et al. 2025) and howler monkeys (Melo et al. 2025), calling this claim into question. There is also evidence that orangutans may experience a similar adrenarche pattern (Prall et al. 2015; Takeshita et al. 2019).

Gorillas, as a close human relative, are prime candidates for the exploration of adrenarche, but have rarely been the subject of such research (Bernstein et al. 2012). Gorillas are known to have a fetal zone (Czekala et al. 1983) and a zona reticularis (Gorilla Doctors, *pers. comm*.), and therefore might experience adrenarche. Promisingly, promotor sequences for the SULT2A1 gene, which produces an enzyme that converts DHEA to DHEAS in the adrenal gland, are identical in humans, chimpanzees, and gorillas, suggesting that DHEAS-related enzymatic activity is likely to be similar amongst these species (Bernstein et al. 2012). However, gorillas’ DHEAS production patterns are poorly described. To the best of our knowledge, there have only been two studies that examined longitudinal changes in DHEAS concentrations in gorillas (Bernstein et al. 2012; Edes 2017). In both studies, the subjects were captive Western lowland gorillas, samples sizes were relatively small, and important age ranges (i.e., young infants) were largely missing. One study found a small rise in DHEAS that peaked around 7–9 years old (Bernstein et al. 2012), while another did not find any relationship between DHEAS and age (Edes 2017). There is still currently no comprehensive characterization of gorilla DHEAS production across their lifespan.

Testosterone (T) is better studied and influential for a much wider taxonomic distribution than DHEAS. T regulates male sexual development and mating-related phenotypes across vertebrates (Norris and Carr 2013a), including birds (Goymann et al. 2007; Lindsay et al. 2011), ungulates (Malo et al. 2009; Preston et al. 2012; McGowan et al. 2018; Gomes Goethlich et al. 2022), canids (Tsutsui et al. 2004a; Rebolledo-Martínez et al. 2026), rodents (reviewed in Bell 2018), felids (West and Packer 2002; Tsutsui et al. 2004b), and reptiles and amphibians (reviewed in Norris and Lopez 2024a, b). In many species, including primates, it plays a key role in male mating-related behaviors and morphological features, including mating effort, aggression, muscle mass, and secondary sexual characteristics (Maggioncalda et al. 1999; Alvarado et al. 2015; Muller 2017; Negrey et al. 2023; Grebe et al. 2024).

Human males experience a spike in T in early infancy, a phenomenon which is dubbed “minipuberty” (Mann and Fraser 1996). Minipuberty is a short-lived activation of the hypothalamus-pituitary-gonadal (HPG) axis in human infants, and is a critical period for the development of later reproductive function (Busch et al. 2023). It is also a period of developmental plasticity that seems to influence important aspects of male development, such as growth rate, pubertal timing, and reproduction/immune tradeoffs (reviewed in Brown et al. 2024). Minipuberty-like high T has been noted in macaques (Nevison et al. 1997; Mann et al. 1998; Plant et al. 2005), marmosets (Dixson 1986; Lunn et al. 1994; Mitchell et al. 2008), chimpanzees (Fuller et al. 1982), mangabeys (Mann et al. 1983), tamarins (Ginther et al. 2002), and perhaps baboons (Gesquiere et al. 2005), indicating it is widespread across the primate order. High postnatal T has also been noted in rats, mice, horses, and sheep (Rhoda et al. 1983; Yu et al. 1983; Corbier et al. 1992).

Other primate species have been shown to follow human-like testosterone patterns with age. For example, in male chimpanzees, T begins to increase roughly between 6 and 9 years old (Young et al. 1993; Seraphin et al. 2008; Wobber et al. 2013; Behringer et al. 2014). This corresponds to increases in body weight and testicular size (Nadler et al. 1987; Marson et al. 1991a), and around the same time, spermatogenesis occurs (Marson et al. 1991b). Male bonobos, on the other hand, show shallower increases in T with development, which may be a product of reduced male mating competition in bonobos relative to chimpanzees (Wobber et al. 2013; but see Behringer et al. 2014). Increases in T with puberty have also been shown in baboons (Gesquiere et al. 2005), mandrills (Wickings and Dixson 1992), marmosets (Abbott and Hearn 1978; Dixson 1986), mangabeys (Mann et al. 1983), and tamarins (Ginther et al. 2002), though one limitation is that in many of these cases, puberty is indirectly inferred by increases in testicular and body size (Plant and Witchel 2006).

Similar to chimpanzees, gorillas exhibit strong male-male competition over mates, both between and within groups (Bradley et al. 2005; Vigilant et al. 2015; Wright et al. 2019). Thus, we expect to see notable increases in T during male gorilla development, and previous research has supported this supposition. In one study with 52 captive Western lowland gorillas (aged 5–40 years), urinary T increased steadily from juvenility into adulthood, and then began to decrease some time before age 20 (Stoinski et al. 2002). In another study with 18 wild mountain gorillas (aged 2.5–26 years), urinary T levels were higher in males ages 10–13 than they were in males < 7 years old (data for 7–10 year old males was not available in this study; Robbins and Czekala 1997). These studies, while extremely informative, were conducted with relatively small sample sizes, and lacked data for key age classes (e.g., infants), meaning that age-related patterns of T production have yet to be thoroughly studied in wild mountain gorillas across the entire range of development.

Here, we draw on a large sample of wild male mountain gorilla fecal samples collected over the full course of the gorilla lifespan to examine how the hormones T and DHEAS change with age. We use enzyme-linked immunosorbent assays (ELISAs) to quantify metabolites of both hormones. While ELISA is a well-established method to measure a variety of hormone metabolites in primates via non-invasive sampling (e.g., Grebe et al. 2023), when measuring androgens one important issue is ensuring that other androgens (i.e., not those that are deliberately being measured) are not causing interference. With T and DHEAS, this is namely each other, as cross-reactivity due to similar chemical structures has been noted in other assays (Möhle et al. 2002; Middle 2007). Thus, we also validate our ELISA results using high-performance liquid chromatography–tandem mass spectrometry analyses (LC-MS/MS) on a subset of the same samples. LC-MS/MS is a precise method of quantifying concentrations of target molecules in a matrix (McDonald et al. 2011). It is an important tool for validating comparatively cheaper and more accessible methods such as ELISAs, which tend to be prone to greater noise and cross-reactivity (Gent et al. 2023). While T ELISAs have previously been validated for use with mountain gorilla feces using other methods (Rosenbaum et al. 2021; Grebe et al. 2023), this is the first time via LC-MS/MS. DHEAS ELISAs have not been previously validated for use with mountain gorilla feces, though other research groups have successfully used similar enzyme immunoassays with macaque (Takeshita et al. 2018; Mouri and Shimizu 2023), hylobatid (Takeshita 2022; Hart et al. 2025), and howler monkey (Melo et al. 2025) feces.

## Methods

### Subjects and samples

The subjects of this study were individually identified, habituated male mountain gorillas monitored by the Dian Fossey Gorilla Fund (DFGF) in Volcanoes National Park, Rwanda. Age class categories were set as: infant = 0–3 years, juvenile = > 3–6 years, subadult = > 6–8 years, blackback = > 8–12 years, silverback = > 12 + years (following Robbins 2011). T was analyzed in samples from 86 individual males aged from 0.09 to 36.4 years (*n* = 31 infants, 20 juveniles, 10 subadults, 30 blackbacks, and 49 silverbacks; mean samples per individual = 27.1, min = 1, max = 89, SD = 26.6). DHEAS was analyzed in samples from 75 individual males (a subset of the individuals analyzed for T) aged from 0.09 to 36.4 years (*n* = 30 infants, 21 juveniles, 14 subadults, 22 blackbacks, and 39 silverbacks; mean samples per individual = 13.0, min = 1, max = 55, SD = 12.6). For both hormones, a single individual may be represented in multiple age-class categories, since the subjects aged across the course of study.

### Sample collection

Opportunistic monthly fecal samples were collected from 2011 to 2024 by trained field staff and scientists who had passed gorilla identification tests. Specific sample collection methods and transport and storage conditions are described extensively elsewhere (Eckardt et al. 2016; Rosenbaum et al. 2021; Grebe et al. 2024). To briefly summarize, fresh samples uncontaminated with rainwater, urine, or other animals’ excreta were taken from the field and labelled with the subject’s name, social group, and date and time of sample collection. Upon returning from the field (on the same day), samples were frozen at -20 °C until being thawed for hormone extraction, generally 2–3 months after collection.

### Hormone metabolite extraction

We used a previously-described, field-friendly hormone extraction method (Eckardt et al. 2016). To summarize, extraction involved weighing out 0.5 ±0.2 g of sample (free from debris such as plant parts), adding 5mL of 90% ethanol, and microhomogenizing the mixture for 1 min. This solution was then poured through a funnel-shaped filter paper to remove the solids. The remaining ethanol was dried down, and the collection tube capped and returned to -20 °C until reconstitution for analysis (Eckardt et al. 2016). Samples were later reconstituted in a commercial assay buffer (Arbor Assays Catalogue #X065) in preparation for ELISA.

### ELISA analysis

We assessed fecal T metabolite concentration in 2,332 fecal samples. Samples were analyzed at three different laboratories over the 13 years that the project encompasses. The majority (*n* = 1,729) of T samples were analyzed using the Arbor Assays ISWE Testosterone Mini-Kit (Catalog #ISWE001) either at the Primate Behavior Lab at the University of Michigan (samples extracted before 2020; *n* = 969), or at the Ellen DeGeneres Campus of the Dian Fossey Gorilla Fund in Rwanda (samples extracted after 2020; *n* = 760). In addition, a subset of T concentration values (*n* = 603) from samples collected between 2011 and 2016 were generated at the Lincoln Park Zoo’s (LPZ) Davee Center for Epidemiology and Endocrinology, using an in-house T assay (antibody R156/7, CJ Munro, UC Davis) that has previously been validated for use with mountain gorilla feces (Rosenbaum et al. 2021). Fecal T measured by the LPZ and ISWE assays is well-correlated in the same sample (Grebe et al. 2023). Parallelisms and other specific T assay information were previously published (Grebe et al. 2023; Rosenbaum et al. 2021).

We assessed fecal DHEAS metabolite concentration in 980 fecal samples using the Arbor Assays DHEAS Kit (Catalog #K054-H5). All DHEAS assays were run at the University of Michigan (*n* = 317 samples; all extracted before 2020) or the Fossey Fund Campus (*n* = 663 samples; extracted post-2020). For DHEAS, we ran a parallelism to assess the ELISA performance of two pools of samples (juvenile and adult males) versus the kit standard, which indicated satisfactory performance (SI Fig. 1).

Since the absolute value of hormone concentrations inevitably varies with analysis location and assay, unless otherwise noted we z-scored all hormone values within analysis location (University of Michigan, Fossey Fund Campus in Rwanda, or LPZ). We centered the mean on 2, rather than 0, so that where appropriate we could use logged z-scores to satisfy assumptions of residual normality.

To minimize potential issues with sample degradation, we only used reconstituted samples that were thawed a total of three or fewer times. The exact number of thaw cycles was only available for samples analyzed in Rwanda. To test whether thaw cycles affected hormone metabolite concentrations, we fit OLS linear models predicting log hormone concentration (ng/g) from thaw cycle (*≤* 3), using the subset of samples for which exact thaw counts were available (*n* = 747 for T; *n* = 650 for DHEAS). These models confirmed that that thaw cycle did not predict logged T concentrations (R^2^ = 0.001, *p* = 0.19, CI [-0.11, 0.02]) nor logged DHEAS concentrations (R^2^ = 0.0007, *p* = 0.22, CI [-0.02, 0.11]).

Only “baseline” samples (i.e., those not known to have been collected after intergroup encounters and/or aggressive interactions that would likely increase T in the short term) were included in our analysis (Rosenbaum et al. 2021). In all locations and for both hormones, only samples with intra-assay coefficients of variation (CVs) of < 15% were included in the analysis. The inter-assay CV of DHEAS assays run in Michigan and Rwanda were 13.1% and 10.6% respectively. The inter-assay CV of T assays in Michigan, Rwanda, and at LPZ were 8.8%, 10.1%, and 9.4% respectively.

### Assay validation

To assess whether our T and DHEAS ELISAs were successfully capturing our molecules of interest, we conducted LC-MS/MS validations at the University of Michigan. We selected 12 previously-extracted and reconstituted samples from 12 individual males spanning the full age range of our subjects (0.09–36.4 years old) for analysis using both the ELISA and LC-MS/MS methods.

Sample preparation for LC-MS/MS analysis of T and other 3-keto-Δ4-steroids followed previously documented procedures (Wright et al. 2020) with the following departures: 100 µL aliquots of sample reconstitute from each processed fecal sample were diluted with 200 µL HPLC-grade water (Fisher Scientific, W5-4) and 100 µL internal standard with known concentrations of deuterium-labeled steroids in 40% LCMS-grade methanol (Fisher Scientific, A456-4). Supported Liquid Extraction (SLE) elution was performed using 1.8 mL of methyl tert-butyl ether (MTBE; Fisher #60-016-45, Honeywell CHROMASOLV). Samples were evaporated in a Savant Speedvac vacuum evaporator (SPD1030) at ambient temperature after the rotor had been chilled at − 20 ºC for at least 3 h, and evaporated samples were kept on ice until reconstituted with 200 µL 40% methanol to prepare for immediate LC-MS/MS analysis.

For DHEAS, the unlabeled and deuterium-labeled steroid standards (DHEAS and DHEAS-d6) were obtained from Cerilliant. A 10 µL aliquot of methanol from the processed fecal sample was diluted with 290 µL HPLC water, followed by the addition of 100 µL of internal standard including DHEAS-d6 (8000 pg/ml) in 40% methanol. Extraction of DHEAS was performed by SLE using Isolute+ 400µL SLE columns (Biotage). Samples were loaded into SLE columns, assisted by 1.0 psi nitrogen for five seconds on a Pressure + 48 manifold. After five minutes, samples were eluted with 1.4 mL butanol (HPLC-grade; Fisher #A383SK-1) and chloroform (Merck SUPRASOLV #1.02432) (1:1) under gravity for 10 min, followed by five seconds of 0.5 psi nitrogen and then another five minutes under gravity. Next, columns were blown dry by gradually increasing nitrogen pressure from 0 to 9.0 psi over the course of about 30 s, and eluant was then evaporated at ambient temperature with a pre-cooled rotor for 1 h under vacuum (Savant Speedvac SPD1030). Samples were kept on ice until they were reconstituted with 200 µL 40% methanol and immediately analyzed.

For LC-MS/MS analysis, 20 µL of sample was injected via autosampler into an Agilent 6490 triple quadrupole mass spectrometer and analyzed using previously documented acquisition and quantitation methods for sulfated steroids (Rege et al. 2016) and 3-keto-Δ4-steroids (Wright et al. 2020). Due to one implausibly high DHEAS value, three samples (the problem sample along with two randomly selected control samples) were re-run. The re-run value for the problem sample and the means of the original and re-run control sample values were retained for further data analysis.

### Comparison of ELISA and LC-MS/MS results

To assess how well correlated our ELISA-derived and LC-MS/MS-derived results were, for each of the two test types, concentrations were converted to ng/g using the mass of each fecal sample and then z-scored to standardize the scales for comparison. We report correlations between ELISA and LC-MS/MS z-scores as Spearman’s rho (a non-parametric rank association; Spearman 1904). In addition, we use Deming regression with the “mcr” package (version 1.3.3; Potapov et al. 2024) in R (version 4.5.1; R Core Team 2025) to evaluate whether ELISA and LC-MS/MS z-scores change in similar proportions to one another (Cornbleet and Gochman 1979). A high correlation together with a Deming slope close to one indicates that our ELISAs are capturing the hormones of interest, and identifying the same patterns of variation found using LC-MS/MS. LC-MS/MS DHEAS hormone concentrations were logged before z-score transformation for visualization purposes and for use in the Deming regression due to the presence of one high outlier.

To assess possible assay cross-reactivity (a common problem with ELISAs that rely on non-invasively collected samples; Gent et al. 2023), we report correlations between LC-MS/MS T and ELISA DHEAS, and vice versa. In addition, we also measured 23 other 3-keto-Δ4-steroids and three other sulfated steroids via LC-MS/MS, and compared them to our T and DHEAS ELISA results via Spearman correlation tests (SI Table 1). We only included steroids that were detected in at least 75% (> 8) of samples using LC-MS/MS. If the correlations between our target hormones (ELISA T and DHEAS) and the other hormones measured via LC-MS/MS are low, this is a promising indication that our assays are not subject to significant amounts of cross-reactivity.

### Statistical analysis of ELISA results

We used generalized additive models (GAMs) to model the effect of age on T and DHEAS z-scores using the “mgcv” package (version 1.9-3; Wood 2011) in R (version 4.5.1; R Core Team 2025). We only included individuals up to 30 years old in the GAM due to sparse data from older individuals (61 samples for T and 9 samples for DHEAS from the same 2 silverback males); visually, we present these data separately with boxplots. We assessed model fit via inspection of normality and heteroscedasticity of residuals, and basis dimension checks using “gam.check” in the “mgcv” package (Wood 2011). Z-scores were logged to satisfy residual normality assumptions, and back-transformed for plotting. Individual ID was included as a random smoothing effect, as not all individuals were sampled equally due to the logistical constraints of collecting samples from wild animals.

Age was included as a main smoothing effect with penalized-splines (P-splines). By default, GAMs are constructed with variable numbers of polynomials that are joined at “knots” (B-splines; Eilers and Marx 1996). P-splines use the same approach, but add an additional penalty for differences between neighboring spline coefficients, helping to prevent over-fitting (Eilers and Marx 1996). We used this more conservative approach because our data could easily be subject to over-fitting due to large variation in data points both within and between individuals.

We then calculated the first derivative of each curve using the “gratia” package (Simpson 2023). We used the derivative to determine the ages with the greatest rates of change in hormone concentrations, and to visualize the ages at which each hormone switched from negative to positive rates of change and vice versa (i.e., the peaks and nadirs). The exact ages of these transitions were taken from the minimum and maximum curve estimates of the original GAM.

## Results

### Assay validations

**Fig. 1:**
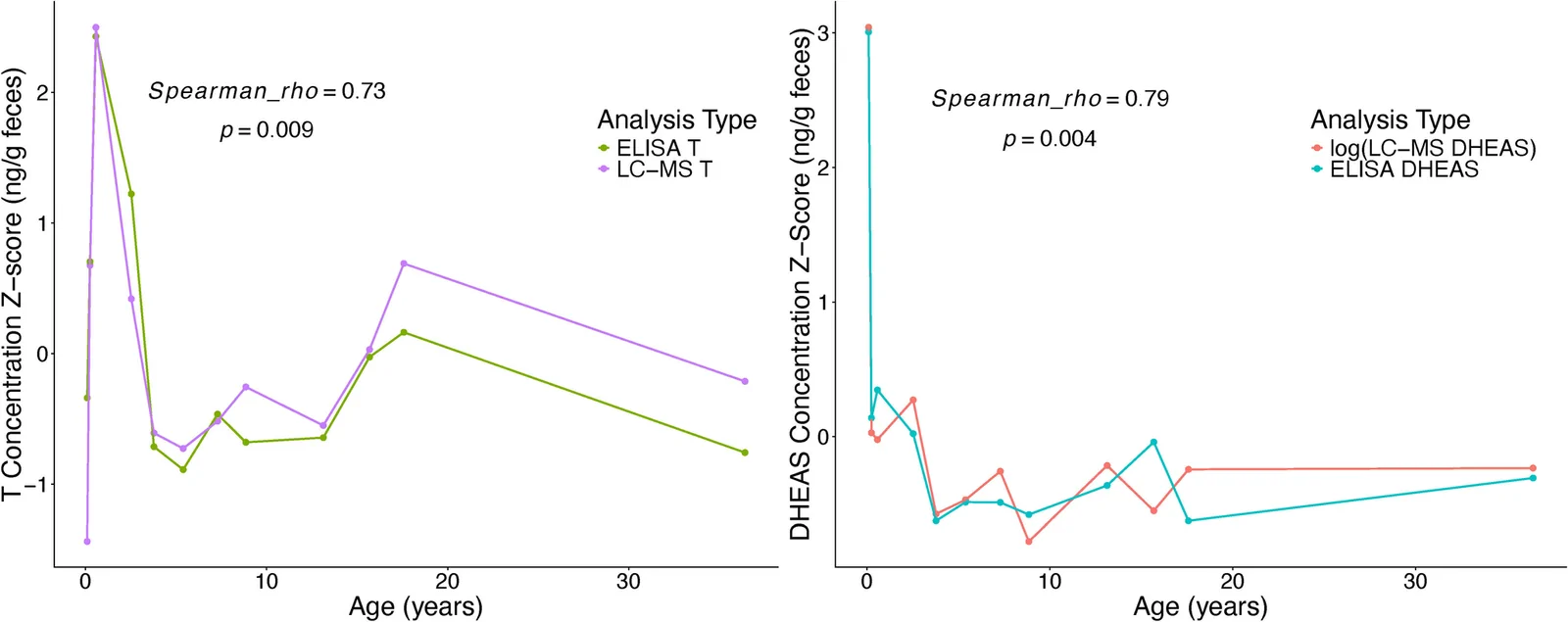
Left: Comparison of T concentrations measured by high-performance liquid chromatography–tandem mass spectrometry (LC-MS/MS) and enzyme-linked immunosorbent assay (ELISA). Right: Comparison of DHEAS concentrations measured by LC-MS/MS and ELISA methods. For both hormones, concentrations were z-scored to standardize measurement scales for comparison. For DHEAS, LC-MS/MS concentrations were logged before z-score transformation for visualization purposes

T LC-MS/MS z-scores strongly correlated to T ELISA z-scores (Spearman’s Rho = 0.73, *p* = 0.009; Fig. 1). Likewise, DHEAS LC-MS/MS z-scores strongly correlated to DHEAS ELISA z-scores (Spearman’s rho = 0.79, *p* = 0.004; Fig. 1). Deming regression further showed that ELISA and LC-MS/MS (logged for DHEAS) z-scores changed proportionally with one another for T (slope = 1, CI [0.48, 1.64]) and DHEAS (slope = 1, CI [0.44, 1.45]).

There were no significant correlations between ELISA T and DHEAS z-scores and the z-scores of any other 3-keto-Δ4-steroids or sulfated steroids measured using LC-MS/MS (SI Table 1). Moreover, LC-MS/MS DHEAS z-scores were not significantly correlated with ELISA T z-scores (Spearman’s rho = 0.57, *p* = 0.055), nor were LC-MS/MS T z-scores correlated with ELISA DHEAS z-scores (Spearman’s rho = 0.20, *p* = 0.54).

### Relationship between T, DHEAS, and male age

**Fig. 2:**
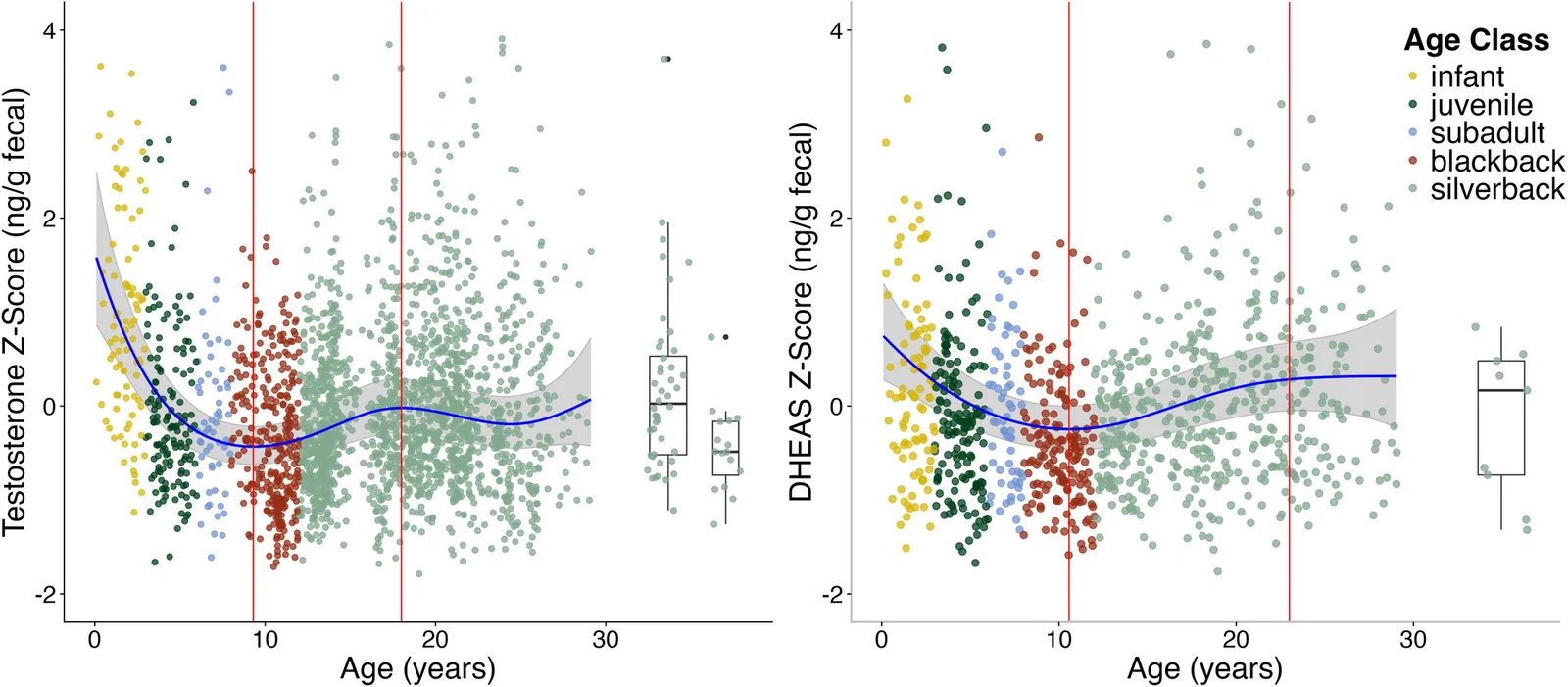
Generalized additive model-generated curves of T and DHEAS z-scores by age. Hormone concentrations were z-scored within analysis location. The effect of age was smoothed with a P-spline, and individual ID was included as a random smoothed effect. Due to relatively sparse data for males over age 30, we used boxplots to visualize results obtained from these individuals. Age class categories are: infant = 0–3 years, juvenile = > 3–6 years, subadult = > 6–8 years, blackback = > 8–12 years, silverback = > 12 years (following Robbins 2011). Red vertical lines represent the ages at which hormones begin to increase and decrease/plateau, and grey bands represent 95% confidence intervals

The GAM-generated curves for T and DHEAS concentrations over age are displayed in Fig. 2. The age splines were significant for both T (deviance explained = 13.7%, edf = 5.33, *p* < 0.001) and DHEAS (deviance explained = 15.7%, edf = 3.6, *p* < 0.001). For both, hormone concentrations were high in infancy, decreased into juvenility and subadulthood, and then increased within the blackback age class (8–12 years old). T began to increase at 9.3 years old while DHEAS, surprisingly, began to increase *afterwards* at 10.7 years old. T then reached a peak at 18 years old, while DHEAS began to plateau at ~ 23 years old.

**Fig. 3:**
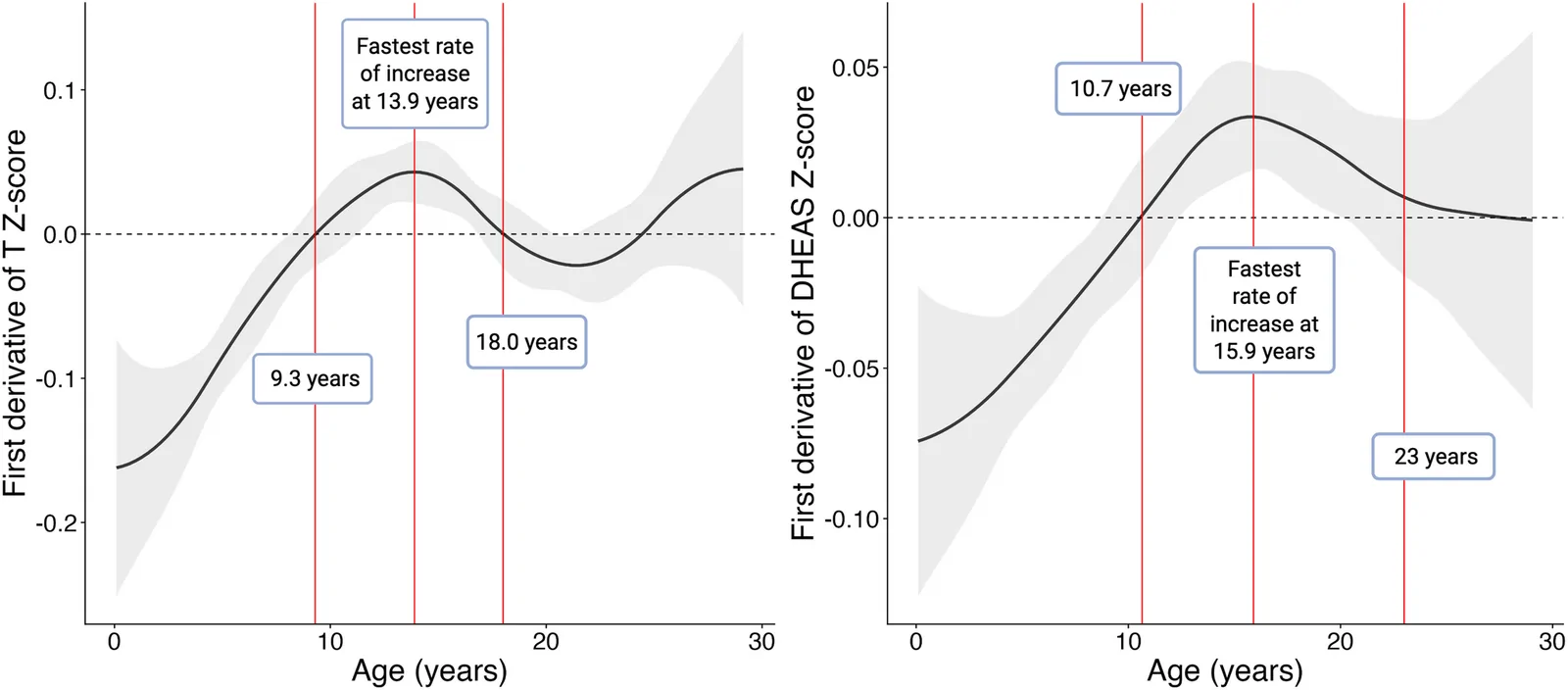
The first derivative of the generalized additive model-generated curves for Testosterone and DHEAS z-scores over age, with major milestones noted. The dashed horizontal line at y = 0 denotes where rates of change switch from positive to negative, and vice versa. The exact ages of these transitions were taken from the minimum and maximum curve estimates in Fig. 2. Gey bands represent 95% confidence intervals. Figure labels were created in Biorender.com

The first derivative of each curve is displayed in Fig. 3. T reached a maximum rate of increase at 13.9 years old, while for DHEAS this occurred at 15.9 years old. Note that the nadirs and peaks from Fig. 2 correspond to the ages at which the rates of change switched from negative to positive and vice versa in Fig. 3.

## Discussion

Here, we have examined T and DHEAS hormone metabolite concentrations in the fecal extracts of wild male mountain gorillas from early infancy to old age. Overall, results indicate that both hormones are high in infancy, decrease into juvenility, and begin to increase within the age category of blackback hood (8–12 years of age; Robbins 2011). These patterns have interesting implications for our understanding of male mountain gorilla development, both in its own right and within the broader context of the evolution of primate developmental trends.

### Assay validation

Our results indicate that our selected T and DHEAS ELISAs are indeed measuring their intended hormone targets. Correlations between ELISA and LC-MS/MS – generated values were very high (Fig. 1), higher than any other hormone measured here (SI Table 1), and the values tended to change proportionally with one another. Moreover, the T LC-MS/MS-generated values were not significantly correlated with the DHEAS ELISA-generated values, nor vice versa, and no other 3-keto-Δ4-steroids or sulfated steroid was significantly correlated with our ELISA results. This lack of relationships is a promising indication that our assays are not subject to meaningful amounts of cross-reactivity.

### Age and DHEAS

In our samples, DHEAS was high in infancy and decreased into juvenility (Fig. 2), similar to patterns observed in humans (Auchus and Rainey 2004), chimpanzees (Sabbi et al. 2020), baboons (Castracane et al. 1981; Ducsay et al. 1991), sooty mangabeys (Mann et al. 1983), and macaques (Kemnitz et al. 2000; Conley et al. 2011). This indicates that gorillas likely have an active fetal zone of their adrenal gland that produces DHEAS up until birth, which then regresses. This is consistent with previous morphological research indicating that gorillas possess a fetal zone of the adrenal gland during the fetal and neonatal stages (Czekala et al. 1983).

Intriguingly, however, male gorillas’ later-life DHEAS pattern seems to deviate from that of two of their closest living relatives, humans and chimpanzees. After a low period during blackback-hood, gorillas’ DHEAS increases when males are between 10 and 11 years old––roughly 1.5 years *after* the increase of T (Fig. 2). From a comparative perspective, this is surprising. Adrenarche is characterized by increases in DHEAS that correspond to the formation of a functioning ZR (Auchus and Rainey 2004). In humans and chimpanzees, this milestone occurs before puberty: in chimpanzees, urinary DHEAS begins increasing at ~ 3 years old (Sabbi et al. 2020), and in humans, significant increases in circulating DHEAS occur ~ 6–8 years old (Auchus and Rainey 2004). Gorillas mature more quickly than chimpanzees, so we would expect their adrenarche, if anything, to occur even earlier than it does in chimpanzees (Bernstein et al. 2012). Yet based on fecal DHEAS data, gorillas’ DHEAS increase is so delayed that it comes *after* the rise in T.

The biological significance of this relatively late rise in DHEAS is not yet clear. Morphological data from the adrenal gland, particularly the formation of the ZR, are needed to confirm when adrenarche truly occurs for gorillas. Until such data are available, we can only speculate that the observed increase in DHEAS is indicative of a newly-developed ZR, and thus adrenarche. We are, however, not the first to document such a delayed rise in DHEAS: in one early study, pig-tailed macaques were reported to show post-pubertal increases in DHEAS as well (Smail et al. 1982). To the best of our knowledge, though, there has been no discussion of or follow-up on this interesting result.

There are very limited data regarding variation in adrenal developmental patterns within primates. The best-known examples are macaques (Nguyen et al. 2008; Conley et al. 2011, 2012) and baboons (Castracane et al. 1981; Ducsay et al. 1991), who also experience adrenarche, but diverge from the human-like pattern. In a rapid time-window in the months after birth, their fetal zones regress and are concurrently replaced by the adult adrenal cortex. Thus, these species have high DHEAS in infancy that continues to fall throughout development (i.e., there is no pre-pubertal increase in DHEAS; see Nguyen and Conley 2008 for a review). Without morphological data, it might be easy to assume that adrenarche does not occur for these species at all, when in fact a version of it does (Conley et al. 2012). Likewise, we must be cautious in what claims can be made about gorilla adrenarche before morphological data can be obtained.

It is also possible that the observed rise in DHEAS is related to T-mediated developmental changes. Adrenarche and puberty are known to be two separate processes, but even in humans, their potential interaction and overlap are not well understood (Brown et al. 2024; Augsburger et al. 2024). In humans, rising levels of adrenal androgens during adrenarche are associated with some of the earliest secondary sexual characteristics (SSCs), such as pubic and auxiliary hair (Rege and Rainey 2012). Moreover, while the majority of circulating T in males is of gonadal origin, DHEA can be converted locally to T within peripheral tissues (Labrie et al. 1998, 2001), making it hard to know how much androgenic activity within these tissues is of adrenal versus gonadal origin. This leaves open the possibility that the observed rises in T and DHEAS are associated with overlapping aspects of development.

Speculatively, the fact that male gorilla DHEAS increases at 10.7 years of age may suggest it has a role in socio-sexual behavioral development, as has been suggested in humans (Hochberg 2009; Del Giudice 2018). DHEAS can act as a neuro-steroid that impacts behavior, activates sex-specific brain pathways (Friess et al. 2000; Del Giudice 2018; Cumberland et al. 2021), and may have a relationship with impulsivity (Aluja et al. 2024) and mood (Fleshner et al. 1997; Noda et al. 2000; Campbell 2006). Increases in DHEAS associated with adrenarche are therefore likely influential in juvenility as we expand our social experiences and skills to prepare for sexual maturation and adulthood (Campbell 2006; Hochberg 2008, 2009; Del Giudice et al. 2017; Cumberland et al. 2021). The process of becoming a fully developed, silverback gorilla likely involves large shifts in sexual, aggressive, and dominance behaviors, as well as the need for new social skills (e.g., securing mating opportunities). It is plausible, therefore that DHEAS may play a similar role in this notable developmental switch as it does in humans. Future research could focus on the relationship between DHEAS and behavioral shifts in the silverback transition.

Our results differ from those derived from a limited sample of captive Western lowland gorilla serum, in which a small rise in DHEAS was detected before age 10 (Bernstein et al. 2012). It is possible that sample matrix differences help explain this discrepancy. At this point, we do not fully understand the process of DHEAS being excreted in feces, and an ideal validation would have involved measuring DHEAS in serum samples as well (e.g., Mouri and Shimizu 2023; Melo et al. 2025). In this instance, however, collecting serum was simply not possible due to the logistical and ethical constraints of studying a highly endangered wild primate. Thus, we cannot yet confidently say that fecal DHEAS is truly representative of circulating levels of DHEAS in gorillas. Regardless, there is strong evidence in other species that adrenal hormones are indeed present in feces (e.g., corticosterone in mice and rats; Cinque et al. 2018; Touma et al. 2004), and the current data support this.

It is also notable that females are missing from our DHEAS analysis, for whom adrenarche may actually be a more influential milestone. Female reproduction, and therefore development, is more constrained than that of males due to the higher costs of gestation and lactation over sperm production (Hochberg 2009; Brown et al. 2024). The timing of adrenarche, therefore, may be more impactful for females than males, as has been suggested with the timing of puberty (Brown et al. 2024). Moreover, although research with humans generally indicates that DHEAS levels are higher in males than females (Goldman and Glei 2007), studies with non-human primates have shown the opposite (marmosets: Pattison et al. 2007, 2009; howler monkeys; Melo et al. 2025). In marmosets, males do not even develop a functional ZR to produce DHEAS, while females do depending on their social/reproductive environment (Pattison et al. 2007, 2009). A full understanding of gorilla adrenarche would therefore also include data from female gorillas.

Later in life for male mountain gorillas, results show that DHEAS then reaches a peak rate of increase at 15.9 years old, and then plateaus. Based on the limited number of samples we have for males age 30+, gorilla DHEAS then decreases into old age (Fig. 2). In humans, male DHEAS levels peak in their 20s, and decrease thereafter in a phenomenon known as adrenopause (Samaras et al. 2013). The exact role of DHEAS in later life remains unknown, but DHEA and DHEAS treatments in elderly patients have revealed potential effects on sexual function, bone health, muscle mass, mobility, and cardiovascular health (reviewed in Samaras et al. 2013). A similar trend––in which DHEAS continues to rise later in life before declining––has been noted in gibbons (Hart et al. 2025), orangutans (Prall et al. 2015), and *Cercopithecus* and *Pan* (Bernstein et al. 2012). It seems, therefore, that male gorillas also experience this decline in adrenal androgen production, though more research would be needed to identify any health-related effects.

### Age and testosterone

Testosterone was highest in early infancy and decreased into juvenility (Fig. 2), indicating that the gorilla HPG axis, similar to humans and other primates, is activated in early life. Thus, gorillas too seem to experience a “minipuberty,” adding to the growing list of primates in which this has been documented. Further research is needed to identify if this period is important for future gorilla reproductive function, as it is for humans (Busch et al. 2023). For instance, research could examine how T levels during minipuberty associate with adult fertility, which would likely involve measures of sperm count (Busch et al. 2023), and/or offspring count.

While evidence for some version of minipuberty may be unsurprising, what is surprising is that the T concentrations in infancy in our samples was extremely high––higher than many adult male samples (Fig. 2), which is generally not seen in male human infants (Andersson et al. 1998) nor other primate species (mangabeys: Mann et al. 1983; marmosets: Dixson 1986; Lunn et al. 1994; tamarins: Ginther et al. 2002; Macaques: Plant et al. 2005). While it is tempting to conclude that this is simply a result of ELISA cross-reactivity, LC-MS/MS results, which do not suffer from the same cross-reactivity issues as ELISAs, also suggest that T is extremely high in infancy. These data raise interesting questions about the hormonal profiles of infant male gorillas; zoo-housed animals may be more feasible subjects for future exploration, given how difficult it is to collect samples from wild infant gorillas. It is rare for a study of wild primates to include samples from such young individuals, so our study is an important first step in characterizing minipuberty-like HPG axis activation in gorillas.

After a period of quiescence in adolescence, T began to increase at 9.3 years of age (Fig. 2). This is around the time that male and female growth trajectories diverge, and around the timing of peak male growth velocity for body length, sagittal crest, and shoulder breadth (Galbany et al. 2017). The fastest rate of T increase was at 13.9 years old (Fig. 3), which is within the age range during which young silverbacks become fully developed. Previous research indicates that males attained maximum body size in different dimensions between the ages of 13.1–16.7 years old (Galbany et al. 2017).

Testes and spermatogenesis data are required to confirm if this period of increasing T captures physiological puberty for gorillas. At least for some males, physiological puberty clearly must come earlier than 9.3 years of age, as one male sired an offspring just before turning 9 years old (Vigilant et al. 2015). There could, therefore, be a slight mismatch between the timing of sexual and physical development. Chimpanzee research also hints at this possibility: in captive males, one study that found urinary T began to increase around 8 years old (Behringer et al. 2014), while another found that spermatogenesis began as early as ~ 6 years old (Marson et al. 1991b), and adult body mass was attained between ~ 12–14 years old (Curry et al. 2023). Even in human males, somatic growth continues after sexual maturity, as spermarche (the first ejaculation containing sperm) begins ~ 13 years of age (Guízar-Vázquez et al. 1992), while adult height is generally not reached until ~ 18 years of age (Tanner 1981). It could be the case, therefore, that our data are not capturing increases in T associated with early sexual function, but rather primarily with physical development. Because both processes involve T and are overlapping in time, separating out the two is difficult.

In gorillas, an important part of physical development is secondary sexual characteristics (SSCs), which include large body size and high sagittal crests, and which have been shown to correspond with T concentrations in other primates specifically (reviewed in Muller 2017) and mammals more generally (e.g., West and Packer 2002; Malo et al. 2009; Gomes Goethlich et al. 2022). For instance, T levels are associated with male mandrill (Setchell et al. 2008) and macaque (Vandenbergh 1965) skin redness, arm circumference (as a proxy for muscle mass) in male baboons (Muehlenbein et al. 2001), and the presence of SSCs in orangutans, which include large body size, cheek flanges, and a throat sack (Kingsley 1988; Marty et al. 2015). Moreover, orangutans in the process of developing SSCs were found to have the highest T concentrations in one study (Maggioncalda et al. 1999), and those that developed SSCs earlier retained higher levels of T later in life (Emery Thompson et al. 2012). Our results, showing that T increases around the time of increased physical growth, therefore, are consistent with known relationships between T and SSC development. Indeed, prior research on wild mountain gorillas documented an increase in urinary T levels in one maturing male before the physical onset of his SSCs (Robbins and Czekala 1997).

Our data indicate that T reaches a peak at ~ 18 years old and declines into later adulthood (Fig. 2). Though there is potentially a small uptick shortly before age 30, given the limited number of samples we have in the ~ 27–30 year age range, it seems most plausible that this is a result of limited sample sizes at older ages. In human males, T begins to decline gradually after about 35–40 years old, and late in life is accompanied by declines in physical and sexual parameters (Wu et al. 2008; Handelsman et al. 2015; Santos and Bhasin 2021). Thus, 18 years old may represent prime gorilla physical and sexual development, though behavioral, mating, and paternity data would be useful to confirm this.

## Conclusion

Our data on fecal T and DHEAS across the lifespan in wild male mountain gorillas suggests both similarities and differences to patterns observed in other apes. T and DHEAS are both high in infancy, which is suggestive of a minipuberty and a fetal zone of the adrenal gland similar to that of humans and many other primates. However, unlike humans, gorillas’ DHEAS levels do not rise again until after their T begins increasing. We need both hormone and morphological data from a wider range of primate species to understand the true range of intra-order variation in primate adrenal development, and how gorillas fit into this broader evolutionary framework.

## Supplementary Information

Below is the link to the electronic supplementary material.

Supplementary Material 1

## Data Availability

The data used for research analysis involves biological samples (fecal samples). The government of Rwanda through the Rwanda Development Board (RDB) requires that publications resulting from biological samples collected within Rwanda need to be first approved by RDB before submission/publication. In addition, biological samples used for a specific research project approved by RDB cannot be used for other research projects before approval by RDB. Due to these regulations, the datasets cannot be made publicly available on a depository platform but can be requested through the Dian Fossey Gorilla Fund and shared upon approval by RDB. The R code used for all data analysis can be found here (https://osf.io/ndfpm/overview? view_only=8fadcb2a7697403f98d58498e5823759).
