# Supplementary material for: Increases in fecal testosterone precede increases in fecal dehydroepiandrosterone in wild male mountain gorillas (Gorilla beringei beringei)
Source: J Comp Physiol B. 2026 Jul 27;196(5):823–37. doi: 10.1007/s00360-026-01702-y (PMC13658407; doi:10.1007/s00360-026-01702-y)
Supplement: Supplementary file 1 [file 360_2026_1702_MOESM1_ESM.docx]

**Supplementary Information**

**SI Fig. 1.** Parallelism plot to compare sample pools (adults and juveniles) versus kit standard performance.

**SI Table 1.** All steroids measured with LC-MS/MS. Only those detected in at least 9 out of 12 samples were included in subsequent analyses. All 3-keto-Δ4-steroids are compared with ELISA T-generated z-scores, and all sulfated steroids are compared with ELISA DHEAS-generated z-scores.

| *3-Keto-Δ4-Steroids* | *Detected in > 8 samples?* | *Spearman’s Rho*  *(p-value)* |
| --- | --- | --- |
| Estriol (E3) | N |  |
| 18-OH-Cortisol | N |  |
| 18-Oxo-Cortisol | N |  |
| 18-OH-Corticosterone | N |  |
| Cortisol | N |  |
| Cortisone | Y | 0.57 (p = 0.06) |
| 21-Deoxy-Cortisol | N |  |
| Estradiol (E2) | N |  |
| Aldosterone | N |  |
| 11-OH-Testosterone | N |  |
| 11-Keto-Testosterone | N |  |
| 11-Deoxy-Cortisol | N |  |
| 11-OH-Androstenedione | N |  |
| 11-Keto-Androstenedione | N |  |
| Corticosterone | N |  |
| 16-OH-Progesterone | N |  |
| Estrone (E1) | N |  |
| Testosterone | Y | 0.73 (p = 0.009) |
| 11-OH-Progesterone | N |  |
| 17-OH-Progesterone | Y | 0.34 (p = 0.29) |
| 11-Keto-Progesterone | N |  |
| Androstenedione-AD | Y | 0.52 (p = 0.08) |
| 11-Deoxy-Corticosterone | Y | 0.41 (p = 0.19) |
| Progesterone | Y | 0.18 (p = 0.57) |
| *Sulfated Steroids* |  |  |
| Androst-5-ene-diol-SO4 | N |  |
| 17-OH-Pregnenolone-SO4 | N |  |
| DHEAS | Y | 0.79 (p = 0.004) |
| Pregnenolone-SO4 | N |  |
|  |  |  |
